# Atopic dermatitis in 32 children treated with dupilumab in real life. A new contribution

**DOI:** 10.1002/ski2.413

**Published:** 2024-07-03

**Authors:** Isabel Betlloch‐Mas, María Armengol‐García, Noelia Jara‐Rico, Ruben Hernández‐Quiles, Laura Berbegal DeGracia

**Affiliations:** ^1^ Department of Clinical Medicine Miguel Hernández University Alicante Spain; ^2^ Department of Dermatology Dr. Balmis de Alicante General University Hospital Alicante Spain; ^3^ ISABIAL Research Institute Alicante Spain

Dear Editor,

There is little experience on the efficacy and safety of dupilumab in clinical practice to treat atopic dermatitis (AD) in childhood. A case series of 71 children and adolescents (≤18 years) with AD treated with dupilumab was recently published.^1^ They were followed for up to 4 years and treatment was effective in most cases. However, treatment failure and side effects in some children and adolescents highlighted the need for new alternatives. We would like to add to the evidence by reporting the efficacy and safety results of a retrospective study carried out at Dr Balmis University Hospital in Alicante, Spain, in children (≤14 years) with a diagnosis of AD who received dupilumab for at least 4 weeks. Treatment with dupilumab was administered according to the technical specifications in cases of moderate or severe AD with indication for systemic treatment. The study was approved by the ethics committee of our hospital as part of a multicenter study and did not consider it necessary to obtain informed consent from patients as it was a retrospective study with anonymized data.

Epidemiological, clinical and treatment variables, comorbidities, severity scales—EASI (Eczema Area Severity Index), SCORAD (SCORing AD), investigator global assessement and body surface area, pruritus and sleep numerical rating scale (NRS), quality of life, drug dose and adverse effects were collected.

Statistical analysis was carried out with the SPSS programme for windows version 27.0. A descriptive study of the sample was carried out with absolute values and percentages for qualitative variables and mean and standard deviation (SD) for quantitative variables. After checking the normality in the distribution of quantitative variables, they were compared with the Student's *t*‐test, and a *p*‐value <0.05 was considered statistically significant.

Table 1 presents the characteristics of our cases (*n* = 32). During follow‐up, the mean initial EASI of 21.2 (SD 10.6) dropped to 8.5 (SD 6.7) at 4 weeks (*p* < 0.001) and 6.1 (SD 4.1) at 12 weeks (*p* < 0.001). At week 4, 11 of the 32 children evaluated (34.4%) achieved EASI 75 (≥75% improvement), a score that increased to 52.2% in the 23 children evaluated at week 12.

**TABLE 1:** Epidemiological and clinical characteristics, severity, treatment and adverse effects in children with atopic dermatitis treated with dupilumab.

Variable	*N* (%) or mean (SD)^a^
Male sex	16 (50%)
Mean age on AD diagnosis, months	16.1 (16.8)
Family history of atopy	9 (28.1%)
Non‐atopic comorbidities	6 (18.8%)
Coeliac disease	1 (3.1%)
Diabetes mellitus	1 (3.1%)
ADHD	1 (3.1%)
Others	3 (9.4%)
Atopic comorbidities	19 (59.4%)
Asthma	16 (50%)
Allergic rhinitis	13 (40.6%)
Food allergy	15 (46.9%)
Predominant pattern of lesions	
Folds	10 (31.1%)
Generalized	22 (68.8%)
Affected areas	
Face	24 (75%)
Trunk	24 (75%)
Folds	32 (100%)
Hands	9 (28.1%)
Previous topical treatment	
Topical corticosteroids	32 (100%)
CNIs (tacrolimus and/or pimecrolimus)	26 (81.3%)
Previous systemic treatment	
Antihistamines	31 (96.9%)
Systemic corticosteroids	31 (96.9%)
≥2 cycles	29 (90.6%)
Cyclosporine	8 (25%)
Methotrexate	5 (15.6%)
Age on initiation of dupilumab, years	8.13 (SD 3.06)
Age group on initiation of dupilumab	
<6 years	9 (28.1%)
6–12 years	16 (50%)
>12 years	7 (21.9%)
Mean initial severity scores	
EASI	21.2 (SD 10.6)
SCORAD	58.1 (SD 16.7)
Pruritus NRS	6.9 (SD 3.8)
Sleep NRS	8.3 (SD 2.1)
Adverse effects	17 (53.1%)
Pain at injection site	13 (40.6)
Conjunctivitis	3 (9.4%)
Infections	5 (15.6)
Facial erythema	3 (9.4%)

Abbreviations: AD, atopic dermatitis; ADHD, attention deficit hyperactivity disorder; CNIs, calcineurin inhibitors; EASI, Eczema Area and Severity Index; *n*, number of cases; NRS, numerical rating scale; SCORAD, SCORing Atopic Dermatitis; SD, standard deviation.

^a^Unless otherwise specified in the variable column.

Improvement was sustained over longer‐term follow‐up: mean EASI was 3.0 (SD 3.1) at week 24 (*p* < 0.001), 5.8 (SD 8.1) at week 52 (p.028) and 1.9 (SD 2.07) at week 100 (p.032). We also observed improvement in the SCORAD index, in pruritus and sleep NRS and in atopic comorbidities. Adverse effects were mild; the most frequent was pain at the injection site (*n* = 13; 40.6%), which led to treatment being suspended in two cases. One child stopped treatment owing to lack of efficacy and another for unknown reasons. The treatment was prescribed according to weight and age, with prior authorization from the biologics committee. Eight children (25%) required treatment optimization (increasing the dosing Interval) because of good response and four (12.5%) required a higher dose.

There is limited real‐world research of dupilumab in children with AD^1^, ^2^, ^3^, ^4^, ^5^ and even less in children under 6 years of age.^5^ Our series was characterized by a wide age range (nine of the children were younger than 6 years), early‐onset AD and predominance of generalized involvement. The majority of children had received previous treatment with oral corticosteroids, according to usual clinical practice, although it is not a requirement for starting dupilumab. On the other hand, the previous use of other systemic drugs in 40.6% of cases was carried out mostly before dupilumab was available. Follow‐up lasted 52 weeks in 13 children and 100 weeks in 8 children. The mean initial EASI was somewhat lower than in other series,^1^, ^2^, ^3^, ^4^, ^5^ since the decision to start treatment was based on pruritus and sleep scales in some cases. In general, improvement was rapid: 34.4% of the children achieved EASI 75 at week 4% and 52.2% at week 12. These findings corroborate the rapidity of the drug's action in a significant number of patients.^2^

Comparing the improvement between the week of treatment initiation and the following weeks, the differences in the reduction of EASI were already significant at week 4 (*p* < 0.001) and were maintained over the following weeks (week 12: *p* < 0.001; week 24: *p* < 0.001; week 52: p.001; week 100: p.002). There was a borderline significant reduction in EASI from week 4 to week 12 (*p* = 0.06) and to week 24 (*p* = 0.07). There was no significant reduction from week 24 to weeks 52 and 100.

Under 6 years of age, 62.5% of children reached PASI 75 at week 4, suggesting that the onset of action of dupilumab may be even faster in this age group, a slightly higher percentage than previously reported.^5^ SCORAD, pruritus and sleep scores dropped in parallel with EASI. We also observed a clinical improvement in asthma and rhinitis, which was confirmed because patients no longer used the drugs they usually needed to control their symptoms. The main limitation of our study is the differing follow‐up across the series, as children were started on dupilumab at different times and some values were missing from clinical records. Regarding adverse effects, we observed few cases of conjunctivitis or facial erythema and frequent occurrence of pain at the injection site, unlike in other series.^1^, ^5^

The small sample size and single‐center nature of our study is one of its main limitations.

In conclusion, our experience provides further evidence for the rapid and sustained efficacy of dupilumab in clinical practice in a series of children aged 4–14 years with moderate‐to‐severe AD. With the exception of pain at the injection site which forced the drug to be discontinued in 2 patients, we found no major safety concerns.

## CONFLICT OF INTEREST STATEMENT

The authors declare no conflicts of interest.

## AUTHOR CONTRIBUTIONS

**Isabel Betlloch‐Mas**: Investigation (equal); supervision (equal); writing—original draft (equal); writing—review and editing (equal). **María Armengol‐García**: Investigation (equal); writing—original draft (equal). **Noelia Jara‐Rico**: Investigation (equal); methodology (equal). **Ruben Hernández‐Quiles**: Investigation (equal); methodology (equal). **Laura Berbegal DeGracia**: Investigation (equal); supervision (equal); visualization (equal); writing–review and editing (equal).

## FUNDING INFORMATION

This article received no specific grant from any funding agency in the public, commercial, or not‐for‐profit sectors.

## ETHICS STATEMENT

The study was approved by the ethics committee of our hospital as part of a multicenter study.

## PATIENT CONSENT

Our study is retrospective and is exempt from informed consent of the patients.

## Data Availability

The data that support the findings of this study are available from the corresponding author upon reasonable request.
